# How Nutrition can help to fight against COVID-19 Pandemic

**DOI:** 10.12669/pjms.36.COVID19-S4.2776

**Published:** 2020-05

**Authors:** Faseeha Aman, Sadia Masood

**Affiliations:** 1 Ms. Faseeha Aman, M.Phil. Human Nutrition & Dietetics, Clinical Nutritionist & Lecturer, Former Intern, Aga Khan University, Centre of advanced studies in health and Technology, Rawalpindi, Pakistan; 2 Sadia Masood, MBBS, FCPS, MHPE. FAIMER Fellow Assistant Professor, Department of Medicine, Aga Khan University Hospital, Karachi, Pakistan

**Keywords:** Nutrition, Immunity, Corona pandemic

## Abstract

Currently Covid-19 pandemic is a leading challenge across the globe. It is mandatory to attain and maintain good nutritional status to fight against virus. Nutritional status of individual is affected by several factors such as age, sex, health status, life style and medications. Nutritional status of individuals has been used as resilience towards destabilization during this COVID-19 pandemic. Optimal nutrition and dietary nutrient intake impact the immune system, therefore the only sustainable way to survive in current context is to strengthen the immune system. There is no evidence found that supplement can cure the immune system except Vit C, which is one of the best way to improve immune system. A proper diet can ensure that the body is in proper state to defeat the virus. However along with the dietary management guidelines the food safety management and good food practices is compulsory. This article explores the importance of nutrition to boost immunity and gives some professional and authentic dietary guidelines about nutrition and food safety to withstand COVID-19.

More than 2,500 years ago, Hippocrates said: “Let *food be thy medicine and medicine be thy food*.” Both nutrient intake and incidence of the disease usually influence the nutritional status particularly of developing nations, where everyone is striving for food.^1^ Inadequate diet and infectious diseases can lead to severe malnutrition. Currently, the COVID-19 pandemic is the leading challenge across the globe, therefore scientists and researchers are attempting to create a specific vaccine for this virus but to no avail so far.^2^ Even if they were able to find the vaccination method, there is a high possibility that other antimicrobial resistant infections will prevail in society. Nutritional status is very important to maintain a strong immune system against the virus.

Certain factors such as lifestyle, age, health status, sex, and medications affect the nutritional status of an individual.^3^ During the COVID-19 pandemic, the nutritional status of individuals has been used as a measure of resilience toward destabilization.^1^ Optimal nutrition and dietary nutrient intake impact the immune system through gene expression, cell activation, and signaling molecules modification. In addition, various dietary ingredients are determinants of gut microbial composition and subsequently shape the immune responses in the body.^3^ Therefore the existing evidence suggests that the only sustainable way to survive in the current situation is to strengthen the immune system. An adequate intake of zinc, iron, and vitamins A, B 12, B6, C, and E is essential for the maintenance of immune function. In the current scenario, COVID-19 has imposed a new set of challenges for the individual to maintain a healthy diet.^4^ The state of self-isolation, lockdown, and social distancing are important measures to flattening the curve of the disease, although these measures have severe repercussions on an individual’s life. The act of confining to one’s home has significant impacts on one’s health, including changes in eating patterns, sleeping habits, and physical activity. It would promote sedentary behaviors that affect mental and physical health and lead to an increased risk of obesity.^5^ Fear and anxiety may also cause changes in dietary habits leading to unhealthy dietary patterns and less desire to eat or with lessened enjoyment during eating.^6^

A balanced diet will guarantee a strong immune system that can help withstand any assault by the virus. There is currently no evidence that any supplement can ‘boost’ our immune system and treat or prevent any viral infections, except Vitamin C.^7^ Vitamin C is one of the major constituents of water soluble vitamins which tends to make a strong immune system. The daily recommended dietary allowance for Vitamin C is 90mg/d for men and 75mg/d for women. In the current situation, it is necessary to be aware of the specific types of food that can improve our immune system in order to combat COVID-19.^8^ Here are some professional and authentic dietary guidelines^9^ to withstand COVID-19:

- Eat fruits daily (guava, apple, banana, strawberry, cantaloupe melon, grapefruit, pineapple, papaya, orange, Longman fruit, blackcurrant, pummelo) with a serving size of two cups (4 servings).
- Eat fresh vegetables (green bell peppers, garlic, ginger, kale, lime, coriander (dried), broccoli, green chili pepper) 2.5 cups of vegetables (5 servings) legumes (beans and lentils).
- Eat whole grains and nuts, 180 g of grains (unprocessed maize, oats, wheat, millet, brown rice or roots such as yam, potato, taro or cassava)
- Use nuts like almonds, coconut, and pistachio.
- Red meat can be eaten once or twice per week, and poultry 2−3 times per week. Use foods from animal sources (e.g. fish, fish, eggs, and milk) and 160 g of meat and beans.
- For snacks, choose fresh fruits and raw vegetables rather than foods that are high in sugar, salt or fat. Avoid irregular snacking.
- Do not overcook vegetables as it leads to the loss of important nutrients such as vitamins and minerals.
- When using dried or canned fruits and vegetables, choose varieties without added sugar or salt.
- Make sure the food is prepared and served at acceptable temperatures (≥72°C for 2 mins).
- Limit the salt intake to five g a day.
- Consume unsaturated fats (found in avocado, fish, nuts, soy, olive oil, canola, corn oil, and sunflower) rather than saturated fats (found in butter, fatty meat, coconut and palm oils, cheese, ghee, and cream).
- Drink 8–10 glasses of water every day. It helps to transport nutrients in the blood, gets rid of waste, and regulates the body temperature.
- Avoid all fizzy, carbonated, concentrated juices, and all drinks which contain sugar.
- Maintain a healthy lifestyle of exercise, meditation, and regular sleep. Adequate sleep will help to support immune functioning.
- Eat at home to avoid contact with other people and try to reduce the chance of being exposed to COVID-19.

A proper diet can help to ensure that the body is in the strongest possible state to battle the virus. The food safety management system must provide food safety officials and workers with proper personal protective equipment to avoid contamination.^10^,^7^ Researchers have found that there is no source of virus contamination via food packaging or food.^8^ However, good food practices are always recommended by following them to minimize the risk of contamination which are as follows:

- Wash vegetables and fruits before eating.
- Wash, rinse, and disinfect objects and surfaces every time before and after use.
- Keep cooked and raw foods separate, as it would prevent the harmful microbes from raw foods spreading to cooked foods.
- Use different chopping boards and utensils for cooked and raw foods to prevent cross-contamination.
- Food service workers should use gloves while preparing a meal.
- Try not to display or sell unwrapped food from the self-service counter.
- Frequently disinfect surfaces which came in contact with customers or workers such as door knobs, counters, grocery carts.

A proper and healthy diet can ensure a robust immune system that can resist any onslaught by the virus. A certain amount of particular nutrient saturates into cells and prevents any kind of nutritional deficiency. Individuals consuming well-balanced diets appear to be safer with better immune systems and lower incidence of chronic diseases and infections. The main objective of this article is to induce healthy dietary habits that help to maintain the physical and mental health of individuals.
